# Satisfaction, Perceived Usefulness, and Therapeutic Alliance as Correlates of Participant Engagement in a Pediatric Digital Mental Health Intervention: Cross-Sectional Questionnaire Study

**DOI:** 10.2196/49384

**Published:** 2023-09-06

**Authors:** Landry Huffman, Darian Lawrence-Sidebottom, Jennifer Huberty, Clare Beatty, Monika Roots, Kurt Roots, Amit Parikh, Rachael Guerra

**Affiliations:** 1 Bend Health Inc Athens, GA United States; 2 FitMinded Inc LLC Phoenix, AZ United States

**Keywords:** service satisfaction, satisfaction, patient-provider, adolescent, child, children, youth, mental health, perceived usefulness, internet-based coaching, coach, coaching, internet-based therapy, collaborative care, digital mental health intervention, mental health, engagement

## Abstract

**Background:**

Although evidence suggests that digital mental health interventions (DMHIs) are effective alternatives to traditional mental health care, participant engagement continues to be an issue, especially for pediatric DMHIs. Extant studies of DMHIs among adults suggest that participants’ satisfaction, perceived usefulness, and therapeutic alliance are closely tied to engagement. However, these associations have not been investigated among children and adolescents involved in DMHIs.

**Objective:**

To address these gaps in extant DMHI research, the purpose of this study was to (1) develop and implement a measure to assess satisfaction, perceived usefulness, and therapeutic alliance among children and adolescents participating in a DMHI and (2) investigate satisfaction, perceived usefulness, and therapeutic alliance as correlates of children’s and adolescents’ engagement in the DMHI.

**Methods:**

Members (children and adolescents) of a pediatric DMHI who had completed at least one session with a care provider (eg, coach or therapist) were eligible for inclusion in the study. Adolescent members and caregivers of children completed a survey assessing satisfaction with service, perceived usefulness of care, and therapeutic alliance with care team members.

**Results:**

This study provides evidence for the reliability and validity of an adolescent- and caregiver-reported user experience assessment in a pediatric DMHI. Moreover, our findings suggest that adolescents' and caregivers’ satisfaction and perceived usefulness are salient correlates of youths’ engagement with a DMHI.

**Conclusions:**

This study provides valuable preliminary evidence that caregivers’ satisfaction and perceived usefulness are salient correlates of youths’ engagement with a DMHI. Although further research is required, these findings offer preliminary evidence that caregivers play a critical role in effectively increasing engagement among children and adolescents involved in DMHIs.

## Introduction

In recent years, digital mental health interventions (DMHIs), such as internet-based coaching and therapy, have emerged as effective and scalable alternatives to in-person mental health care. However, low participant engagement, especially among children and adolescents, continues to limit the therapeutic benefits of DMHIs [1-3]. Should these issues of engagement remain unaddressed, DMHIs may lose their long-term viability as mental health treatments for young people. As such, the identification of factors associated with participant engagement is paramount for DMHIs, enabling the tailoring of interventions to maximize therapeutic effects for participants.

Extant studies of DMHIs among adults suggest that participants’ satisfaction, perceived usefulness, and therapeutic alliance are closely tied to engagement. DMHI participants who experience low satisfaction, often due to a complicated or dysfunctional user experience, are more likely to drop out prematurely [4,5]. Conversely, participants who report higher usefulness of a DMHI, such as improvements in their well-being and mental health symptoms, often show increased engagement [1,6,7]. Finally, therapeutic alliance, or the working relationship between participants and care providers, is an often-identified predictor of compliance and engagement in traditional therapeutic interventions, such as face-to-face therapy [8,9]. Nascent evidence suggests positive but inconsistent links between therapeutic alliance and engagement in DMHIs, highlighting the need for further study [10]. As such, understanding how satisfaction, perceived usefulness, and therapeutic alliance contribute to DMHI engagement among children and adolescents is crucial to the continued effectiveness of digitally delivered mental health care.

To address these gaps in extant DMHI research, the purpose of this study was to (1) develop and implement a measure to assess satisfaction, perceived usefulness, and therapeutic alliance among children and adolescents participating in a DMHI and (2) investigate satisfaction, perceived usefulness, and therapeutic alliance as correlates of children’s and adolescents’ engagement in the DMHI.

## Methods

### Design and Treatment

Members (ages 2-17 years) enrolled in care with Bend Health, Inc by December 7, 2022, who had completed at least one care session were eligible for inclusion in the study. Survey data were collected between December 2022 and January 2023. Members and their caregivers were invited via email (sent up to 5 times to each member) to complete a survey assessing the following: satisfaction with service, perception of the usefulness of care, and therapeutic alliance with care team members. Members were given a US $5 electronic gift card upon completion of the survey.

Treatment with the DMHI, Bend Health, Inc, has been described in detail previously [11,12]. In brief, each member has a behavioral care manager (BCM) who coordinates and oversees their care and, in synchronous virtual sessions, coaches or licensed therapists deliver care plans based on cognitive behavioral therapy, behavioral activation, parent management training, mindfulness-based cognitive therapy, motivational interviewing, and mindfulness-based stress reduction. Members may also have sessions with a prescriber. Members may attend up to 5 synchronous virtual sessions per month with their BCMs, coaches, therapists, or prescribers.

### Ethics Approval

Study procedures were approved by an independent institutional review board, Biomedical Research Alliance of New York (BRANY IRB; study identification number 23-12-010-1374).

### Survey

The survey was designed based on prior work assessing therapeutic alliance and service satisfaction among youths participating in digital mental health services [13-15]. The survey assessed the following domains: satisfaction with service, perceived usefulness, and therapeutic alliance. Satisfaction (5 items) assessed the acceptability of the DMHI interface and care services. Perceived improvement (5 items) assessed perceptions of improvements in emotions, emotional skills, and relationships. Therapeutic alliance (12 items) assessed members’ feelings of hope and optimism after sessions, being understood by their care team, and alignment of goals and tasks. Responses were on a 5-item Likert scale (ranging from “strongly disagree” to “strongly agree”). All items are listed in Table S1 and Figures S2-S4 in Multimedia Appendix 1. Caregivers of children (ages 2-12 years) responded to the survey on behalf of their child (caregiver report). Adolescents (ages 13-17 years) responded to the survey on behalf of themselves (self-report).

### Analysis

To determine whether the subscales’ respective items measured the same underlying construct, we conducted an exploratory factor analysis, followed by 3 confirmatory factor analyses (one per subscale). All factor analyses were conducted in R 4.2.0 (R Core Team) using the lavaan package [16,17]. After establishing the dimensionality of subscales, Cronbach alphas were computed for each subscale to determine reliability. Due to power limitations, mean values of subscale items were used for subsequent analyses (maximum mean score 5).

For initial descriptive statistics, survey respondents were differentiated by caregiver report and self-report. Caregiver-reported and self-reported survey scores were compared for each subscale by paired 2-tailed *t* tests. Each responding member’s duration in care was assessed as the number of months between their first synchronous session with a Bend Health care provider (eg, BCM, coach, or therapist) and their survey completion date. Descriptive statistics of care with Bend Health, Inc are reported for 2022. Survey respondents’ demographic information and patterns of participation were then compared to those who were sent the survey but did not respond using paired *t* tests and chi-square tests.

Finally, correlation analyses were used to assess whether there is a significant positive association between engagement (eg, duration of care and the number of total sessions with a coach or therapist) and subscale scores. Each subscale was assessed independently.

## Results

### Respondents Versus Nonrespondents

Of the 213 members eligible for the study, 52 (24.1%) responded (caregiver report: n=33 and self-report: n=19). Comprehensive demographics for all groups are reported in Table 1. Chi-square tests suggested no significant demographic differences between respondents and nonrespondents (Table S5 in Multimedia Appendix 1). Respondents completed the survey, on average, 2.92 (SD 1.39) months after they began care with the DMHI and had completed an average of 3.62 coaching or therapy sessions prior to responding (Table 2). Nonrespondents had completed significantly fewer coaching or therapy sessions (Table 2).

**Table 1:** Demographic information for caregiver-reported, self-reported, and nonrespondent groups.

Demographics			Caregiver report (children; n=33)		Self-report (adolescents; n=19)		Nonrespondents (n=161)
Age (years), mean (SD)			8.0 (2.3)		14.4 (1.2)		11.0 (3.7)
**Sex, n (%)**							
	Female	20 (60.6)		17 (89.5)		88 (54.7)	
	Male	13 (39.4)		2 (10.5)		72 (44.7)	
**Gender or sex conformity, n (%)**							
	Conforming	38 (100)		18 (94.7)		159 (98.8)	
	Nonconforming	0 (0)		1 (5.3)		2 (1.2)	
**Race or ethnicity, n (%)**							
	Asian	1 (3)		1 (5.3)		0 ( 0)	
	Black or African American	0 (0)		0 (0)		1 (0.6)	
	Hispanic or Latino	2 (6.1)		1 (5.3)		14 (8.7)	
	Other or multiracial	8 (24.2)		10 (52.6)		6 (3.7)	
	Unknown	0 (0)		0 (0)		0 (0)	
	White	22 (66.7)		7 (36.8)		55 (34.2)	

**Table 2:** Participation measures for respondents versus nonrespondents.

Participation measure	Respondents (n=52), mean (SD)	Nonrespondents (n=166), mean (SD)	Comparison	
			*t* test (*df*)	*P* value
Total BCM^a^ sessions	1.3 (1.2)	0.9 (1.2)	–2.4 (211)	.02
Total coaching sessions	2.1 (3.2)	1.2 (2.3)	–2.0 (68.3)	.05
Total therapy sessions	1.0 (1.3)	1.0 (1.5)	–0.1 (211)	.94
Total coaching and therapy sessions	3.1 (3.1)	2.2 (2.5)	–2.1 (75.2)	.04
Total prescriber sessions	0.9 (0.9)	0.6 (0.9)	–2.1 (211)	.04

^a^BCM: behavioral care manager.

### Measurement Model and Reliability

Using exploratory factor analysis, we identified a 3-factor solution as most appropriate (Multimedia Appendix 1). This solution aligned closely with our expected subscales of satisfaction, perceived usefulness, and alliance, with the exception of 2 items from the alliance subscale that loaded onto the satisfaction factor and 3 items from the alliance subscale that loaded onto the perceived usefulness factor (Figures S1-S3 in Multimedia Appendix 1 present more details).

**Figure 1:**
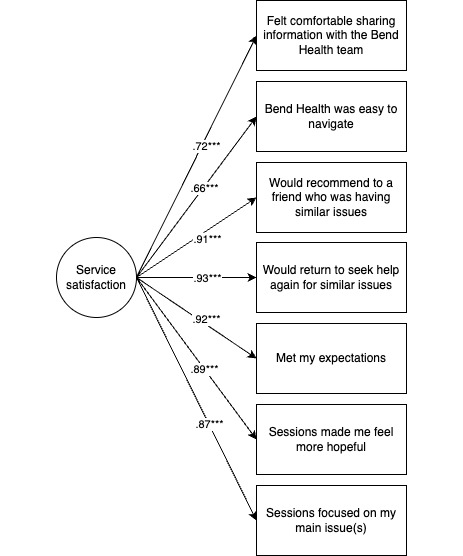
Satisfaction latent factor model (model parameters=14). Model fit is satisfactory (*χ*214=42.7; *P*<.001; comparative fit index [CFI]=0.936; Tucker-Lewis index [TLI]=0.904; standardized root mean square residual [SRMR]=0.039). ***P<.001. Of note, items are abbreviated in the figure, and when necessary, include only the adolescent version. Table S1 in Multimedia Appendix 1 presents the full items.

**Figure 2:**
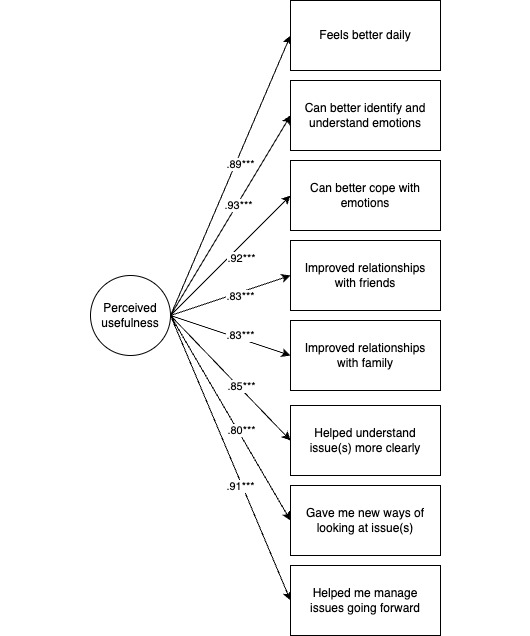
Perceived usefulness latent factor model (model parameters=16). Model fit is satisfactory (*χ*220=25.1; *P*=.19; comparative fit index [CFI]=0.990; Tucker-Lewis index [TLI]=0.987; standardized root mean square residual [SRMR]=0.026). ***P<.001. Of note, items are abbreviated in the figure, and when necessary, include only the adolescent version. Table S1 in Multimedia Appendix 1 presents the full items.

**Figure 3:**
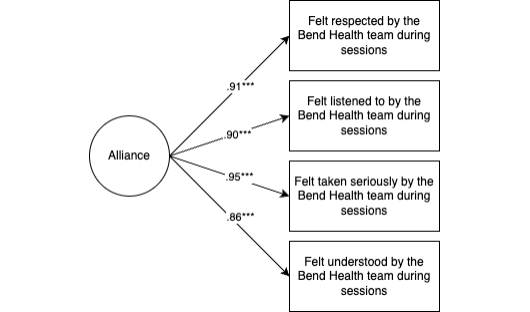
Alliance latent factor model (model parameters=8). Model fit is satisfactory (*χ*22=1.6; *P*=.44; comparative fit index [CFI]=1.000; Tucker-Lewis index [TLI]=1.004; standardized root mean square residual [SRMR]=0.010). ***P<.001. Of note, items are abbreviated in the figure, and when necessary, include only the adolescent version. Table S1 in Multimedia Appendix 1 presents the full items.

Using confirmatory factor analysis, we then confirmed this solution using 3 separate 1-factor models (a 3-factor model was not feasible due to lower power [18]). All models showed acceptable fit based on the comparative fit index (CFI), Tucker-Lewis index (TLI), and standardized root mean square residual (SRMR) fit indices (CFI>0.90; TLI>0.90; and SRMR<0.08 [19]). Factors were highly correlated with each other (alliance with satisfaction=0.90; *P*<.001; alliance with perceived usefulness=0.805; *P*<.001; and satisfaction with perceived usefulness=0.847; *P*<.001). The results of the final measurement models were then used to create mean subscales (Figures 1-3; mean values of each subscale are found in Table S4 in Multimedia Appendix 1). For each subscale, Cronbach alpha was calculated to assess reliability. All subscales indicated high reliability (alliance α=.96, satisfaction α=.95, and perceived usefulness α=.97).

### Correlation of Survey Scores and Engagement

**Figure 4:**
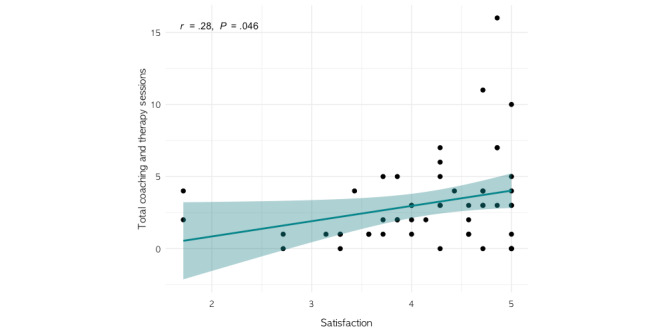
Correlation between satisfaction and total coaching and therapy sessions.

**Figure 5:**
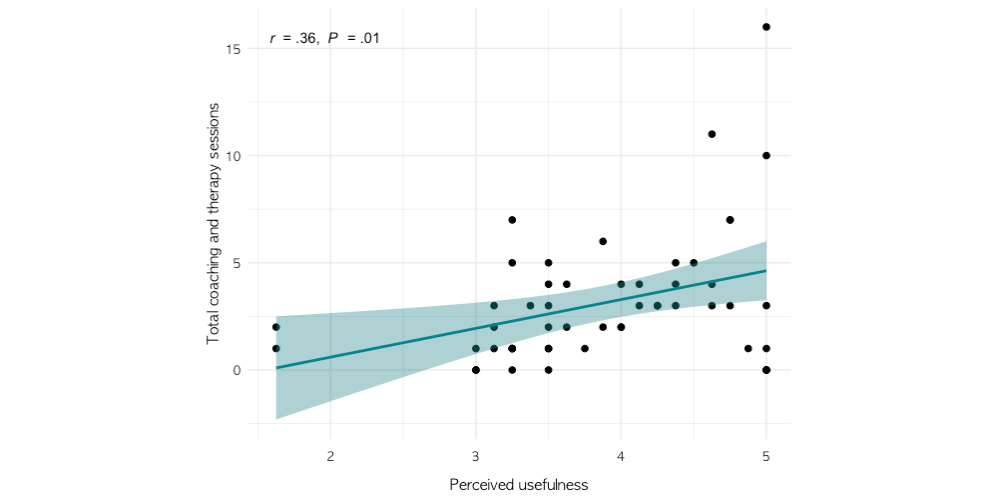
Correlation between perceived usefulness and total coaching and therapy sessions.

No significant associations were found between survey scores and care duration. However, satisfaction and perceived usefulness scores were both significantly correlated with the total number of therapy or coaching sessions (satisfaction: *r*=0.28; *P*=.046 and perceived usefulness: *r*=0.36; *P*=.01; Figures 4 and 5). Coefficients and *P* values of all correlation analyses are included in Table S6 in Multimedia Appendix 1.

## Discussion

### Principal Results

By surveying adolescent members and caregivers of children engaged in care with a DMHI, this study (1) developed a novel measure to assess satisfaction, perceived usefulness, and therapeutic alliance among children and adolescents participating in a DMHI and (2) investigated member-reported satisfaction, perceived usefulness, and therapeutic alliance as correlates of care duration and total sessions with a care provider. Results from our factor analyses revealed that several items intended to assess therapeutic alliance were more closely correlated with members’ satisfaction and perceived usefulness of the DMHI. We found that satisfaction and perceived usefulness were positively linked to total care sessions attended, while therapeutic alliance was not significantly associated with either measure of engagement. These findings suggest that caregivers’ and youths’ positive feelings about the functionality and utility of the services provided may be central to youths’ engagement in the DMHI.

Our final measurement models showed good psychometric properties of a caregiver- and self-reported user experience measure characterized by dimensions of satisfaction, perceived usefulness, and therapeutic alliance. These findings are largely consistent with those by Rickwood et al [15], who identified a similar 3-factor model characterized by service satisfaction (including practical aspects of the web-based and in-session user experience as well as members’ likelihood of recommending and returning to the DMHI), potential outcomes (including the emotional, behavioral, and relational changes associated with participation in the DMHI), and session satisfaction (including elements of the therapeutic alliance, such as feeling listened to and understood). This study largely replicates those findings and builds upon them by not only establishing similar dimensionality using caregiver-reported data but also exploring the subscales’ correlation with indices of engagement.

Compared to satisfaction and therapeutic alliance, we found that perceived usefulness was most strongly associated with the total number of coaching and therapy sessions attended by children and adolescents. Several extant meta-analyses and systematic reviews have identified perceived usefulness as a particularly salient predictor of engagement among adults [1,3,6]. These results suggest a similar trend in the context of a pediatric DMHI, namely that caregivers’ and youths’ beliefs about experiencing tangible change due to the intervention are more important for engagement than their perceptions about the practical user experience and their relationship with the care team practitioners. Although further study is necessary, findings like these offer insights for digital health companies that can inform the development and design of the user experience, which may in turn facilitate engagement, improve patient symptoms, and increase revenue generation.

### Limitations

This study has several limitations. Given the cross-sectional nature of the study, we cannot assess the directionality of these effects. As such, it may be that increased care sessions lead to higher levels of satisfaction and perceived usefulness. Our previous studies have indeed demonstrated time-dependent improvements in mental health outcomes of children and adolescents as well as improvements in the well-being of their caregivers, who are participating in coaching and therapy with Bend Health, Inc [11,12,20]. Future research is necessary to determine whether caregivers’ satisfaction and perceived usefulness improve concurrently with their children’s mental health symptoms over the course of care with a pediatric DMHI.

In addition, the survey completion rate was quite low, limiting our sample size and consequent power to investigate and detect group-level differences in reporter, care type, and symptom type. This issue also highlights the difficulty of voluntary data collection among DMHI participants [21-23]. Future studies should explore methods for more successfully incentivizing DMHI participants to complete optional assessments aimed at improving the user experience. Moreover, given that our survey used convenience sampling of active members only, it is unclear how our survey results are impacted by social desirability bias or selection effects. A random sampling of both active and nonactive members would mitigate this bias and, thus, better characterize users’ experiences.

### Conclusions

Notwithstanding these limitations, this study provides valuable preliminary evidence that adolescents' and caregivers’ satisfaction and perceived usefulness are salient correlates of youths’ engagement with a DMHI. As mental health care becomes more digitalized, the development and standardization of user experience assessments will become increasingly central to DMHI product design, quality assurance, and revenue generation. Our findings speak to this growing need by providing preliminary evidence for the reliability, validity, and implications of a multidimensional user experience assessment administered in the context of a pediatric DMHI.

## Appendix

Multimedia Appendix 1 Survey responses and factor analysis details.

## Abbreviations

BCM: behavioral care manager
CFI: comparative fit index
DMHI: digital mental health intervention
SRMR: standardized root mean square residual
TLI: Tucker-Lewis index

## Data Availability

The data sets analyzed during this study are not publicly available, as this would violate the privacy policy of Bend Health, Inc. However, the aggregated and anonymized data that are not associated with individual users and do not include personal information are available from the corresponding author upon reasonable request.
