# Supplementary material for: Satisfaction, Perceived Usefulness, and Therapeutic Alliance as Correlates of Participant Engagement in a Pediatric Digital Mental Health Intervention: Cross-Sectional Questionnaire Study
Source: JMIR Form Res. 2023 Sep 6;7:e49384. doi: 10.2196/49384 (PMC10512110; doi:10.2196/49384)
Supplement: Multimedia Appendix 1 [file formative_v7i1e49384_app1.docx]

# Supplemental Materials

Table S1. Original survey items

| **Subscale** | **Caregiver version** | **Adolescent version** |
| --- | --- | --- |
| Alliance | My child's sessions made them feel more hopeful | My sessions made me feel more hopeful |
|  | My child's sessions made them feel more optimistic | My sessions made me feel more optimistic |
|  | My child felt respected by our Bend Health team during their sessions | I felt respected by my BendHealth team during our sessions |
|  | My child felt listened to by our Bend Health team during their sessions | I felt listened to by my BendHealth team during our sessions |
|  | My child felt not judged by our Bend Health team during their sessions | I felt not judged by my BendHealth team during our sessions |
|  | My child felt taken seriously by our Bend Health team during their sessions | I felt taken seriously by my BendHealth team during our sessions |
|  | My child felt understood by our Bend Health team during their sessions | I felt understood by my BendHealth team during our sessions |
|  | My child's sessions focused on their main issue(s) | My sessions focused on my main issue(s) |
|  | My child's sessions helped us understand their issue(s) more clearly | My sessions helped me understand my issue(s) more clearly |
|  | My child's sessions gave us new ways of looking at their issue(s) | My sessions gave me new ways of looking at my issue(s) |
|  | The skills my child learned in sessions have helped them to manage their issue(s) going forward | The skills I learned in my sessions have helped me to manage my issue(s) going forward |
|  | The skills my child learned in sessions have helped them to accomplish the changes that they wanted | The skills I learned in my sessions have helped me to accomplish the changes that I want in my life |
| Service satisfaction | My child was able to feel comfortable sharing information with Bend Health | I felt comfortable sharing information with my BendHealth team |
|  | Bend Health was easy to navigate (i.e., easy to log onto sessions, navigate the app) | BendHealth was easy to navigate (i.e., easy to log onto sessions, navigate the app) |
|  | I would recommend Bend Health to a friend whose child was having similar issues to my child | I would recommend BendHealth to a friend that was having similar issues to my own |
|  | If I were to seek help again for my child's similar issues, I would return to Bend Health | If I were to seek help again for similar issues, I would return to BendHealth |
|  | Bend Health met our expectations for what therapy would be like and what my child would accomplish | BendHealth met my expectations about the kind of help I wanted |
| Perceived usefulness | My child feels better day-to-day | I feel better day-to-day |
|  | My child can better identify and understand their emotions | I can better identify and understand my emotions |
|  | My child can better cope with their emotions | I can better cope with my emotions |
|  | My child has improved relationships with their friends | I have improved relationships with my friends |
|  | My child has improved relationships with their family | I have improved relationships with my family |

**Measurement model and reliability**

Prior to conducting the EFA, inter-item correlations were calculated to determine whether any pairs of items were correlated above .90 (suggesting inadequate distinction between items). Three pairs of items exhibited over-correlation (see below), and the second item of each pair was removed from the measure and subsequent analyses.

Table S2. Over-correlated items

| **Retained Item** | **Trimmed Item** | **Correlation** |
| --- | --- | --- |
| My sessions made me feel more hopeful* | My sessions made me feel more optimistic | .92, p < .0001 |
| I felt listened to by my BendHealth team during our sessions | I felt not judged by my BendHealth team during our sessions | .91, p < .0001 |
| The skills I learned in my sessions have helped me to manage my issue(s) going forward | The skills I learned in my sessions have helped me to accomplish the changes that I want in my life | .96, p < .0001 |

*Note. Only the adolescent version is listed, but both adolescent and caregiver items included in the correlation analysis and trimmed from the measure.


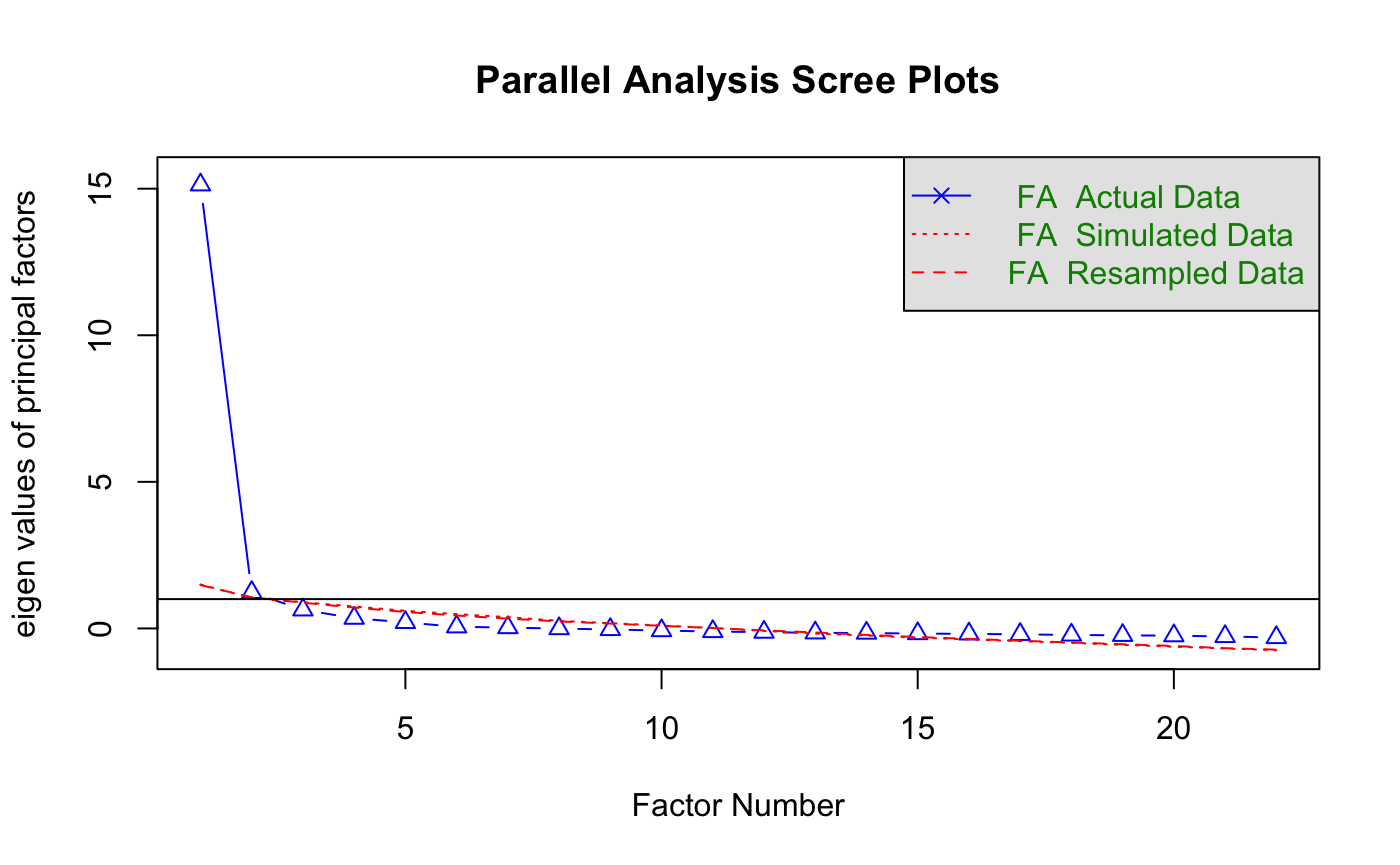


Figure S1. Scree plot indicating the optimal solution at 2-3 factors.

Table S3. EFA 2- and 3-factor solutions

|  | 2-factor solution | | 3-factor solution | | |
| --- | --- | --- | --- | --- | --- |
| Item | Factor 1 | Factor 2 | Factor 1 | Factor 2 | Factor 3 |
| Alliance.1 | .46 | .45 |  | .77 |  |
| Alliance.2 | .37 | .51 |  | .69 |  |
| Alliance.3 |  | .98 |  |  | .83 |
| Alliance.4 |  | 1.00 |  |  | .87 |
| Alliance.5 |  | 1.12 |  |  | 1.03 |
| Alliance.6 |  | 1.03 |  |  | .83 |
| Alliance.7 | .37 | .56 | .36 |  | .42 |
| Alliance.8 | .36 | .54 |  | .62 |  |
| Alliance.9 | .66 |  | .50 | .36 |  |
| Alliance.10 | .54 | .33 | .51 |  |  |
| Alliance.11 | 1.11 |  | .94 |  |  |
| Alliance.12 | 1.15 |  | .90 | .31 |  |
| Satisfaction.1 |  | .61 |  | .49 | .32 |
| Satisfaction.2 | .33 |  |  | .71 |  |
| Satisfaction.3 |  | .69 |  | .57 | .34 |
| Satisfaction.4 |  | .80 |  | .75 | .34 |
| Satisfaction.5 | .57 | .34 |  | .84 |  |
| Usefulness.1 | .83 |  | .74 |  |  |
| Usefulness.2 | .84 |  | .78 |  |  |
| Usefulness.3 | .99 |  | .88 |  |  |
| Usefulness.4 | .67 |  | .68 |  |  |
| Usefulness.5 | .70 |  | .86 | -.35 | .32 |

After investigating EFA output of both 2 and 3 factor solutions, we identified the 3 factor solution as most appropriate, given that it aligned with our anticipated factor structure and had no items that cross-loaded within .05 of each other.

The 3-factor solution aligned closely with our expected subscales—satisfaction, perceived usefulness, and alliance—with a few exceptions. Specifically, 2 items from the alliance subscale loaded onto the satisfaction factor (“My/my child’s sessions made me/them feel more hopeful,” “My/my child’s sessions focused on my/their main issue(s)”), and 3 items from the alliance subscale loaded onto the perceived usefulness factor (“My/my child’s sessions helped me understand my/my child’s issue(s) more clearly,” “My/my child’s sessions gave me new ways of looking at my/their issue(s),” “My/my child’s sessions have helped me/them manage my/their issues going forward”). As such, the alliance factor consisted of the four remaining alliance indicators (see Figures 1 through 3 in main text).

CFA was then used to confirm the EFA solution. Separate factor models were conducted for each factor due to power constraints. All indicator loadings were greater than .65, and all factors showed acceptable fit based on CFI, TLI, and SRMR fit indices (CFI > .90, TLI > .90, SRMR < .08). Factors were highly correlated with each other (alliance ~ satisfaction = .90, p < .000; alliance ~ perceived usefulness = .805, p < .000; satisfaction ~ perceived usefulness = .847, p < .000).

Latent factors were not included in subsequent analyses due to power constraints; rather, the results of the final measurement model were used to create mean subscales. For each subscale, Cronbach’s alpha was calculated to assess reliability. All subscales indicated high reliability: alliance a = .96, satisfaction a = .95, perceived usefulness a = .97.

**Results of measure**

Members in this study reported high satisfaction and therapeutic alliance, with group trends indicating agreement with the survey statements. Perceived improvement scores were slightly lower than the other subscales. On all three surveyed subscales, the caregiver-report group had higher scores than the self-report (adolescent) group. However, between-group comparisons revealed that these differences were statistically significant only for the perceived usefulness subscale (p < .05, Table 3).

Table S4. Scores for each domain for the caregiver-report and self-report groups, and results of the statistical comparison between-reporters.

|  | **All**  (n = 52) | **Caregiver-report**  **(children)**  (n = 33) | **Self-report (adolescents)**  (n = 19) | **Comparison** |
| --- | --- | --- | --- | --- |
| Alliance | 4.62 ± 0.56 | 4.73 ± 0.48 | 4.42 ± 0.65 | t = 1.55, p = 0.13 |
| Satisfaction | 4.16 ± 0.80 | 4.30 ± 0.76 | 3.93 ± 0.83 | t = 1.57, p = 0.12 |
| Perceived usefulness | 3.89 ± 0.81 | 4.10 ± 0.64 | 3.52 ± 0.94 | t = 2.13, p = .04 |

Figures 4 through 6 show proportions of Likert scale responses per each survey item. Most members agreed that they were comfortable sharing their information with the Bend Health Inc. care team (88%; Figure 4). Approximately two in three agreed that they had improved relationships with their family (68%) and 63% agreed that they felt better day-to-day (Figure 5). Nearly all members agreed that they felt respected and taken seriously by their care team (98% and 96%, respectively; Figure 6).


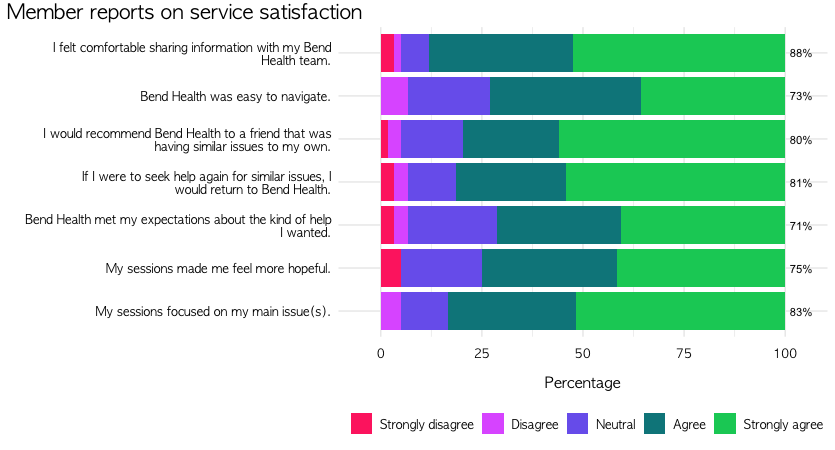


Figure S2. Distribution of responses to the service satisfaction domain items. Percentages to the right of the graph indicate the proportion of “Agree” and “Strongly agree” responses.


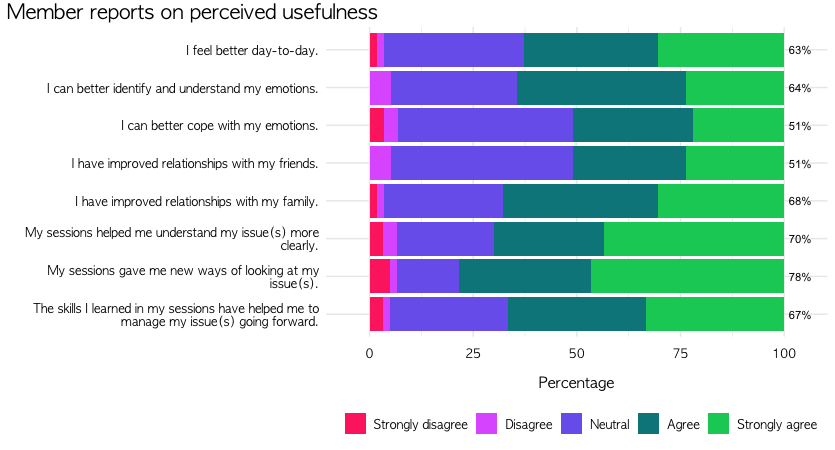


Figure S3. Distribution of responses to the perceived improvements domain items. Percentages to the right of the graph indicate the proportion of “Agree” and “Strongly agree” responses.


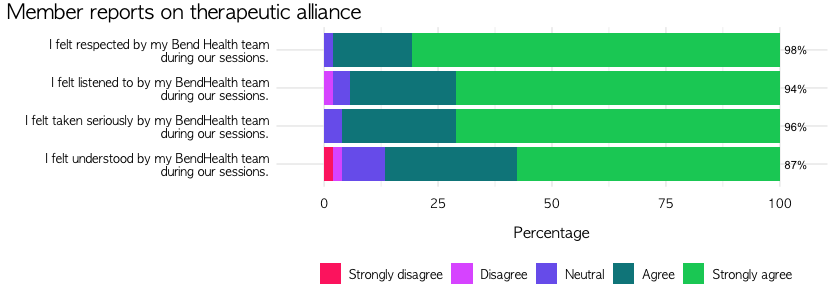


Figure S4. Distribution of responses to the therapeutic alliance domain items. Percentages to the right of the graph indicate the proportion of “Agree” and “Strongly agree” responses.

**Respondents versus non-respondents**

Respondents completed the survey on average 2.92 ± 1.39 months after members began care with the DMHI (Range: 0.56–6.03 months). In 2022, 50% of members had coaching (n = 26; 0.52 ± 0.76 sessions/month), 46.2% had therapy (n = 24; 0.4 ± 0.6 sessions/month), and 9.6% had coaching and therapy (n = 5; 0.92 ± 0.82 sessions/month). Over half of members attended at least one session with a prescriber (55.8%; n = 29; 0.27 ± 0.32 sessions/month) and with a BCM (67.3%; n = 35; 0.34 ± 0.31 sessions/month). Respondents completed significantly more BCM, coaching, and prescriber sessions than non-respondents (see Table 2 in manuscript).

Respondents and non-respondents did not differ significantly based on demographic factors (e.g., age, race/ethnicity, and gender).

Table S5. Statistical comparisons of respondents and non-respondents’ demographic factors.

| Measure | Test | Test value, *P* |
| --- | --- | --- |
| Age | Paired t-test | t = 1.25, *P* = 0.21 |
| Race/ethnicity | Chi-squared test | 𝝌^2^ = 2.01, *P* = 0.73 |
| Gender | Chi-squared test | 𝝌^2^ = 4.57, *P* = 0.10 |

Table S6. Results of all correlation analyses

| Correlated items | Pearson’s *r*, *P* |
| --- | --- |
| Satisfaction ~ duration of care | *r* = .12, *P* = .41 |
| Perceived usefulness ~ duration of care | *r* = .20, *P* = .15 |
| Therapeutic alliance ~ duration of care | *r* = .004, *P* = .98 |
| Satisfaction ~ total coaching/therapy sessions | *r* = .28, *P* = .046 |
| Perceived usefulness ~ total coaching/therapy sessions | *r* = .36, *P* = .01 |
| Therapeutic alliance ~ total coaching/therapy sessions | *r* = .25, *P* = .07 |
